# High-fat diet in pregnant rats and adverse fetal outcome

**DOI:** 10.1080/03009734.2019.1604588

**Published:** 2019-05-07

**Authors:** Parri Wentzel, Ulf J. Eriksson, Emilio Herrera

**Affiliations:** a Department of Medical Cell Biology, Uppsala University, Uppsala, Sweden;; b Department of Biochemistry, CEU San Pablo University, Madrid, Spain

**Keywords:** Animal model, fetal anomaly, fetal outcome, high-fat diet, pregnancy, rodent

## Abstract

**Background:** Although pregestational obesity has been associated with increased risk of adverse fetal outcome, the mechanisms behind are not known. We aimed to investigate the influence of the maternal metabolic state on fetal outcome in rats exposed to either a high-fat diet (HFD) or a control diet (CD). We also investigated the impact of serum collected from HFD/CD pregnant rats on CD embryonic development in whole-embryo cultures.

**Material and methods:** On gestational day 0, 9, 10, or 20 maternal plasma/serum samples were collected as pregnancies were terminated for the estimations of maternal metabolic state and embryo-fetal development. We measured embryonic gene expression of ROS scavenger enzymes as well as genes involved in inflammation in maternal adipose tissue.

**Results:** In HFD maternal plasma/serum, concentrations of glucose, β-hydroxybutyrate, branched-chain amino acids, and leptin were increased, whereas those of triacylglycerol, cholesterol, and palmitic, oleic, linoleic, and α-linolenic acids were decreased. Gene expression of CuZnSOD, IL-6, IL-10, and resistin was increased in HFD maternal adipose tissue, whereas that of CuZnSOD and MnSOD was decreased in HFD-exposed embryos. HFD caused retention of most fatty acids in the maternal liver as well.

**Conclusion:** HFD alters the maternal metabolic state, increases fetal resorptions *in vivo*, and increases the rate of fetal/embryonic malformations both *in vivo* and *in vitro*. These findings suggest that metabolic disturbances in HFD pregnant rats have profound adverse developmental effects in the offspring.

## Introduction

Maternal nutrition is known to play a key role in fetal health (1). The prevalence of maternal overweight/obesity is currently increasing and is associated with an enhanced risk of congenital anomalies (2), such as skeletal, heart, neural tube, and orofacial defects (3,4). In addition, fetal and neonatal complications are more common (5) as well as adverse metabolic postnatal outcomes in the offspring of overweight/obese mothers (6), leading to increased risk of obesity, insulin resistance, and type 2 diabetes later in life (7,8).

The exact mechanisms behind the obesity-induced congenital anomalies are not known, but it is likely that obesity-induced dysmorphogenesis is the result of an interplay between genetic and environmental factors. The latter factors include metabolic alterations in mother and offspring, alterations that may induce developmental changes via (epi)genetic processes.

A number of metabolic alterations have been identified in obese pregnant women. For instance, in obese humans and rodents given a high-fat diet (HFD) during pregnancy and lactation poor glycemic control has been identified (9,10), as well as increased susceptibility to hypertension (11) and changes in hypothalamic gene expression (12). All these changes were associated with adverse outcome in the offspring. Furthermore, in rodents HFDs have been found to induce obesity, and there was a positive relationship between the level of fat in the diet and body weight gain (13). However, pregnant rats on HFD reduce their caloric intake by reducing their intake of energy-dense diet and deriving more calories from fat (14). In rats, HFD throughout pregnancy reduced the concentration of docosahexaenoic acid (22:6 n-3, DHA) and caused a compensatory increase in arachidonic acid (20:4 n-6, AA) in liver lipids of newborn pups (15). Other metabolic factors like branched-chain amino acids (16) and oxidative stress (17) were modified in the offspring of HFD pregnancies, illustrating the capacity of maternal HFD to induce epigenetic and teratogenic effects in the offspring (18). Indeed, oxidative stress, endoplasmic reticulum (ER) stress, and chronic inflammation have also been suggested to be involved in the obesity-induced maldevelopment of the offspring (6,19–21).

In order to contribute to the understanding of obesity-induced teratogenesis, we designed an animal model to study the relationship between HFD-altered maternal metabolism and adverse fetal outcome.

## Material and methods

### Animals

Fifty-eight female rats, 3 weeks old, from a local outbred Sprague-Dawley strain (Biomedical Center, Uppsala University, Sweden), were housed with free access to pelleted food and water in a room maintained at 22 °C with a 12-h light–dark cycle. One half of the rats was fed a high-fat diet (HFD) (35 g% fat, 26 g% protein, 26 g% carbohydrate; D12492, Research Diets Inc., New Brunswick, NJ, USA), while the other half was maintained on a control diet (CD) (4.3 g% fat, 19.2 g% protein, 67.3 g% carbohydrate; D12450B, Research Diets Inc.). The fatty acid (FA) concentrations in CD and HFD were measured. The highest ratio difference between the two diets (i.e. HFD/CD ratio) was found for myristic acid (14:0, MA), followed by stearic acid (18:0, SA), palmitoleic acid (16:1 n-7, POA), oleic acid (18:1 n-9, OA), palmitic acid (16:0, PA), linoleic acid (18:2 n-6, LA), and α-linolenic acid (18:3 n-3, ALA) (Supplementary Table 1, available online).

**Figure 1.**
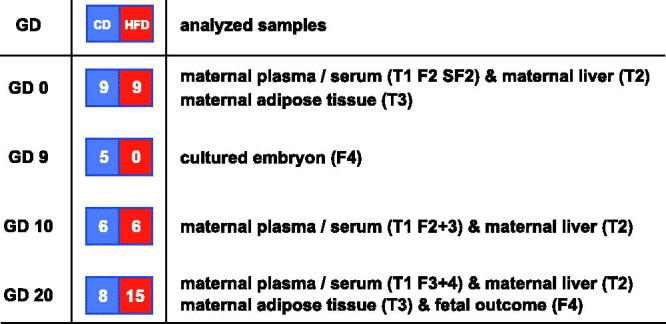
Layout of study. The gestational day (GD) when the different pregnancies are interrupted is displayed in the first column. The number and types of pregnant rats (CD: control diet; HFD: high fat diet) are displayed in the second column. The samples analyzed are displayed in the third column. T1, 2 and 3 denote Tables 1–3, whereas F2, 3, 4 and SF2 denote Figures 2–4 and Supplementary Figure 2 available online.

Three-week-old female pups were fed HFD or CD *ad libitum*, and 9 weeks later (i.e. at 12 weeks of age) the HFD and CD female rats were mated overnight with CD males. Pregnancy was confirmed by a positive vaginal smear next morning (gestational day 0). On gestational day 0 tail vein blood was collected for plasma (in EDTA-Na2 tubes) and serum samples from nine rats of each group. Plasma/serum was separated from fresh blood by centrifugation at 1500 ×*g* for 15 min at 4 °C. Gestational day-0 rats were subsequently killed by cervical dislocation after mild ether anesthesia, and samples of maternal liver and adipose tissue were secured (Figure 1).

During pregnancy rats were maintained on their respective diet. Pregnancy was interrupted on gestational day 9 in five CD rats for the production of embryos aimed for whole-embryo culture (WEC). In gestational day-10 rats from the two dietary groups, pregnancy was interrupted for embryonic mRNA evaluation (six HFD and six CD rats) and collection of maternal liver. Also, on gestational day 10, plasma samples (in EDTA-Na2 tubes)/serum samples from maternal tail vein blood were collected and prepared as described above. Pregnancy was interrupted in 15 HFD rats and 8 CD rats on day 20 by cervical dislocation after mild ether anesthesia after collection of tail vein blood for plasma/serum samples, as above. Fetuses were dissected out from each uterine horn and evaluated with regard to fetal and placental weight (data not shown), malformations, and resorptions. Furthermore, maternal liver and abdominal adipose tissue were collected and kept at -80 °C until analysis.

In addition, from the same HFD and CD rats (gestational day 20), maternal blood was collected from the abdominal aorta, immediately centrifuged and prepared *ad modum* New, (22) and stored at −80 °C until used in whole-embryo culture.

### Ethical evaluation

All animal procedures were performed according to the Guide for the Care and Use of Laboratory Animals (NIH, 1985) and were approved by the Animal Ethics Committee of the Medical Faculty of Uppsala University.

### Skeletal staining

Whole 20-day-old fetuses were fixed in 95% ethanol. Staining of the skeleton was performed with Alcian Blue and Alizarin Red for 24 h at 37 °C, followed by clearing of the soft tissue by submersion in 1% KOH for 48 h (23).

### Whole-embryo culture (WEC)

Pregnant CD rats were killed by cervical dislocation after mild ether anesthesia on gestational day 9. Embryos in their intact yolk sacs were dissected out and cultured in serum collected from HFD or CD day-20 pregnant rats, diluted to 80% with saline (22). The embryos were incubated in Falcon 50 ml tubes (4–5 embryos/tube) in a roller incubator at 60 rev/min for 48 h at 38 °C. After culture, the embryos were dissected out of their yolk sacs and examined and scored under a stereomicroscope. A malformation score of 0 indicated a completely normal embryo; a score of 1 indicated a single minor deviation. A score of 5 denoted one major malformation, whereas a score of 10 indicated an embryo with multiple major malformations such as open neural tube, rotational defects, and/or heart enlargement. Furthermore, we determined crown–rump length and somite number of each embryo for estimation of embryonic growth (24).

### Analyses in plasma/serum, diets, and tissues

Plasma glucose, triacylglycerols (TAG), cholesterol, and non-esterified fatty acids (NEFA) were determined enzymatically using commercial kits (glucose, TAG, and cholesterol: Spinreact Reactives, Girona, Spain; NEFA: Wako Chemicals, Neuss, Germany). Insulin was analyzed by an enzyme-linked immunoassay (Mercodia, Uppsala, Sweden), and β-hydroxybutyrate was analyzed in serum (Cobas MIRA^®^ multichannel analyzer, Hoffman-La-Roche, Basle, Switzerland) using a standard reagent kit (Hoffman-La-Roche; Boehringer Mannheim, Germany; and Sigma).

Nonadecanoic acid (19:1) (Sigma Chemical Co., St. Louis, MO, USA) was added as the internal standard to fresh aliquots of frozen diet, frozen plasma, frozen liver, and frozen adipose tissue, which were used for lipid extraction and purification in chloroform-methanol (2:1) containing 0.005% BHT (25). The final lipid extract was evaporated to dryness under vacuum and the residue resuspended in methanol/toluene and subjected to methanolysis in the presence of acetyl chloride at 80 °C for 2.5 h as previously described (26). Fatty acid methyl esters were separated and quantified on a Perkin-Elmer (Waltham, MA, USA) gas chromatograph (Autosystem) with a flame ionization detector and a 20 mm × 0.25 mm Omegawax capillary column (Merck, Kenilworth, NJ, USA). Nitrogen was used as carrier gas, and the fatty acid methyl esters were compared with purified standards (Sigma Chemical Co.). Quantification of the fatty acids in the sample was performed as a function of the corresponding peak areas compared with that of the internal standard. Liver lipid extracts were also used for the analysis of TAG and cholesterol as described (27) and phospholipids (Spinreact Reactives, Barcelona, Spain). Branched-chain amino acids in serum were analyzed chromatographically after deproteinization with sulphosalicylic acid using a Biochrom 30 (Biochrom, Cambridge, UK) with norleucine as internal standard. Adiponectin, leptin, and resistin were measured in serum by an enzyme-linked immunosorbent assay (ELISA) (EMD Merck Millipore, Burlington, MA, USA).

### Sum of fatty acids

Sum of saturated fatty acids (SFA) and monounsaturated fatty acids (MUFA) represents four different fatty acids. Sum of polyunsaturated n-6 fatty acids (PUFA) and polyunsaturated n-3 fatty acids (PUFA) represents seven and five fatty acids, respectively. In addition, we chose to present some of the fatty acids individually, which are important with regard to embryonic development.

### HOMA determination

HOMA-IR was estimated in day-0 pregnant rats on CD (*n* = 9), or HFD (*n* = 9) by using the formula:

(1) HOMA-IR=([glucose]×[insulin])/405

where plasma samples were expressed in mg/dL (glucose) and µU/mL (insulin).

### Gene expression

Total RNA and cDNA from each embryo were isolated with a RNeasy mini kit (Qiagen, Hilden, Germany), and each embryo was lysed in 350-µL buffers followed by DNase treatment (RNase-free DNase; Qiagen). Isolated total RNA was washed twice with 50 µL RNase-free water, and the accumulated flow-through was collected, yielding the total RNA sample. Lastly, 1 µL of an RNase inhibitor (RNA Guard; Amersham Pharmacia, Uppsala, Sweden) was added to each sample. One microgram of total RNA was used for complementary DNA (cDNA) preparation. For cDNA synthesis, we used first-strand beads (Ready To Go; Amersham Pharmacia Biotech). The resulting cDNA was diluted threefold with RNase-free water. One microliter of cDNA purified from each embryo with 10 ng of converted total RNA was amplified and measured in duplicate with real-time PCR using the MyIQ Optical Thermal Cycler (Bio-Rad Laboratories AB, Sundbyberg, Sweden) with SYBR® Green Supermix (Bio-Rad Laboratories) used to detect the PCR product. Specific primers for CuZnSOD, MnSOD, Gpx1, TNF-α, IL-6, IL-10, adiponectin, and resistin were constructed with the aid of the Primer3 free software (available at http://primer3.sourceforge.net/) and subsequently purchased from TIB Mol-Biol (Berlin, Germany; Supplementary Table 2, available online). We have previously assessed the stability of expression of various housekeeping genes and found the glucose-6-phosphate dehydrogenase (G6Pdh) gene to be constant in day-10 and day-11 embryos (data not shown). Therefore, we chose the G6Pdh gene as a reference in the RT-PCR protocol regardless of maternal metabolic state.

Controls were included in each run of the RT-PCR assay. For each primer pair, one sample with no cDNA (with only RNase-free water) was included. To exclude the possibility of remaining DNA fragments being present in the samples, 10 ng of the total RNA of each sample were amplified in the MyIQ Optical Thermal Cycler. No PCR product was found in the water or the total RNA samples. Furthermore, we excluded the avian myoblastosis virus-RT enzyme in the cDNA preparation and found no amplified PCR product.

Results were analyzed for each sample with relative quantification comparing the difference between sample and reference crossing point (Cp) values. The relative abundance of mRNA/G6Pdh in each sample was determined using the following equation to yield the ratio (R) sample to G6Pdh:

(2) $$R=2^{-({\mathrm{Cp}}_{\mathrm{sample}}-{\mathrm{Cp}}_{G6P\mathrm{dh}})}$$

Total RNA from freshly frozen samples (in liquid nitrogen) of maternal abdominal adipose tissue was isolated using AurumTM Total RNA Fatty and Fibrous Tissue Kit (Bio-Rad Laboratories, Inc., Hercules, CA, USA). One mL of PureZOL was added to each sample and immediately homogenized and then centrifuged for 5 min. Then 0.2 ml chloroform was added to the supernatant, followed by incubation at room temperature for 5 min. Thereafter the samples were centrifuged again for 15 min, and the upper aqueous phase was carefully transferred to a new tube and an equal amount of 70% ethanol was added and then mixed thoroughly. The mixture was then shifted to the spin column and washed with 700 µL of low-stringency wash solution, treated with DNase 1, washed again with 700 µL high-stringency and then low-stringency solution before finally eluting total RNA with elution solution.

### Statistical evaluation

The aim of the study was to analyze the effect of HFD on fetal outcome. The rate of adverse outcome (malformations and resorptions) was therefore chosen as primary endpoint. Comparisons between different experimental groups were based on individual rats (maternal parameters) or litter means (fetal parameters), and values are expressed as mean ± SEM. Statistical comparison between two groups was performed with one-way ANOVA with Fisher’s least significant difference (LSD) as *post hoc* test. The rates of malformations were compared with chi-square statistics. A value of *P* < 0.05 was considered to denote a statistically significant difference.

## Results

At the start of the experiment, the body weights in the 3-week-old pups did not differ between the groups. However, after 9 weeks on the HFD or CD, there was a slight weight difference between the HFD and CD groups on gestational day 0 (Supplementary Figure 1, available online). This difference remained throughout pregnancy (data not shown).

### Plasma/serum components

The glucose concentration was higher in HFD rats than in CD rats, whereas the insulin concentration was similar (Table 1). HOMA calculations reveal no difference between the two groups (Supplementary Figure 2, available online).

**Table 1.** Plasma components/fatty acid concentrations in day-0, day-10, day-20 pregnant rats on control diet (*n* = 9, 6, 8), or high-fat diet (*n* = 9, 6, 15).

Diet and day of pregnancy	CD 0	CD 10	CD 20	HFD 0	HFD 10	HFD 20
Glucose (mg/dL)	123 ± 2			136 ± 3^d^		
Insulin (µg/L)	0.7 ± 0.1			0.9 ± 0.2		
NEFA (µmol/L)	332 ± 15	402 ± 37	705 ± 91^b^^,^^c^	323 ± 18	391 ± 32	1082 ± 130^b^^,^^c^^,^^d^
TAG (mg/dL)	47 ± 4	82 ± 5	222 ± 34^b^^,^^c^	37 ± 4^d^	45 ± 5^d^	147 ± 25^b^^,^^c^^,^^d^
Cholesterol (mg/dL)	67 ± 4	71 ± 3	101 ± 11^b^^,^^c^	38 ± 4^d^	49 ± 5^d^	129 ± 7^b^^,^^c^^,^^d^
β-Hydroxybutyrate (µmol/L)		58 ± 6			109 ± 8^d^	
Sum SFA	713 ± 14	696 ± 47	1422 ± 98^b^^,^^c^	620 ± 55	646 ± 23	1023 ± 44^b^^,^^c^^,^^d^
14:0 MA	9 ± 1	12 ± 1	28 ± 3^b^^,^^c^	5 ± 1	4 ± 1^d^	8 ± 1^d^
16:0 PA	391 ± 21	375 ± 30	1032 ± 81^b^^,^^c^	268 ± 28	255 ± 17	580 ± 26^b^^,^^c^^,^^d^
18:0 SA	295 ± 11	293 ± 16	350 ± 15^b^^,^^c^	334 ± 29	370 ± 9^d^	425 ± 20^b^^,^^c^^,^^d^
Sum MUFA	244 ± 23	328 ± 29	933 ± 84^b^^,^^c^	201 ± 47	197 ± 32	456 ± 43^b^^,^^c^^,^^d^
18:1 (n-9) OA	214 ± 19	284 ± 25	826 ± 71^b^^,^^c^	190 ± 45	185 ± 32	433 ± 41^b^^,^^c^^,^^d^
Sum n-6 FA	991 ± 17	754 ± 38^a^	1420 ± 81^b^^,^^c^	836 ± 73	881 ± 41	1228 ± 59^b^^,^^c^^,^^d^
18:2 (n-6) LA	437 ± 16	335 ± 22	894 ± 62^b^^,^^c^	283 ± 33^d^	268 ± 3	460 ± 38^b^^,^^c^^,^^d^
18:3 (n-6) GLA	15 ± 4	15 ± 2	22 ± 3^c^	9 ± 2	13 ± 1	18 ± 3^b^
20:4 (n-6) AA	500 ± 27	369 ± 18^a^	388 ± 18^b^	491 ± 40	538 ± 21^d^	591 ± 32^b^^,^^d^
Sum n-3 FA	84 ± 3	91 ± 5	182 ± 11^b^^,^^c^	74 ± 4	75 ± 3	196 ± 10^b^^,^^c^
18:3 (n-3) ALA	16 ± 2	12 ± 2	47 ± 5^b^^,^^c^	7 ± 2^d^	6 ± 1	9 ± 2^d^
22:5 (n-3) DPA	16 ± 2	12 ± 1	24 ± 2^b^^,^^c^	14 ± 1	14 ± 2	17 ± 3^d^
22:6 (n-3) DHA	44 ± 3	50 ± 4	93 ± 6^b^^,^^c^	50 ± 3	51 ± 3	163 ± 11^b^^,^^c^^,^^d^

Fatty acid concentrations are expressed as mg/L. Mean ± SEM.

a *P* < 0.05, day 0 versus day 10, for each group.

b *P* < 0.05, day 0 versus day 20, for each group.

c *P* < 0.05, day 10 versus day 20, for each group.

d *P* < 0.05, CD versus HFD at the corresponding day of pregnancy.

CD: control diet; HFD: high-fat diet.

In both HFD 20 and CD 20 rats the plasma NEFA concentrations increased during pregnancy, and they were higher in the HFD 20 rats than in the CD 20 rats (Table 1).

The plasma TAG and cholesterol concentrations increased from day 0 to day 20 of pregnancy in both groups. However, the plasma TAG concentrations were lower in the HFD rats than in the CD rats at all time points measured. The plasma cholesterol concentrations in the HFD 0 and HFD 10 rats were lower than in the corresponding CD pregnant rats, whereas on gestational day 20 the HFD cholesterol concentration exceeded that of the CD rats. In the HFD rats the maternal serum β-hydroxybutyrate concentration was higher when compared with that of CD rats on day 10 of pregnancy (Table 1).

At day 20 of pregnancy, the sum of SFA was lower in HFD rats than in CD rats. In addition, plasma concentrations of myristic (14:0, MA) and palmitic acid (16:0, PA) were lower in HFD rats than in CD rats. However, the concentration of stearic acid (18:0, SA) was higher in the former (Table 1).

The sum of MUFA and the concentration of oleic acid increased from day 0 to day 20 in both groups. Moreover, at gestational day 20 the sum of MUFA and the concentration of oleic acid in HFD rats were lower than in the corresponding CD rats. The sum of PUFA n-6 FA was decreased in HFD 20 rats, when compared with CD 20 rats (Table 1).

Plasma linoleic acid (18:2 n-6, LA) concentrations were lower in both day-0 and day-20 pregnant HFD rats than in the corresponding CD rats, whereas there were no differences between the groups with regard to γ-linoleic acid (18:3 n-6, GLA). In contrast, the AA (20:4 n-6) plasma concentration was higher in the HFD 10 and HFD 20 rats compared with that of the CD 10 and CD 20 rats. In both groups, the sum of PUFA n-3 FA increased to the same extent during pregnancy (Table 1). Plasma concentrations of α-linolenic acid (18:3 n-3, ALA) were lower in HFD 0 and 20 rats compared with CD rats on corresponding days. Plasma concentration of docosapentaenoic acid (22:5 n-3, DPA) was lower in the HFD group on day 20. In contrast, the concentration of DHA (22:6 n-3) in HFD 20 rats was increased compared with the concentration in CD 20 rats.

**Figure 2.**
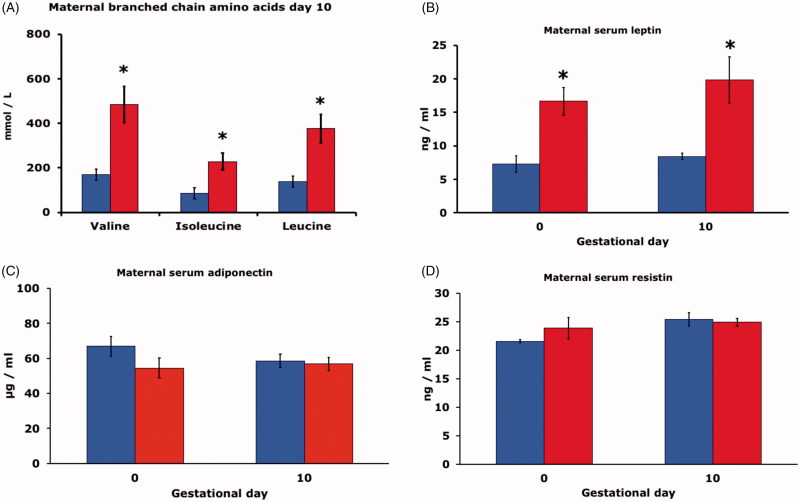
(A) Serum branched chain amino acid concentration (mmol/l) of pregnant rats on control diet (CD, blue bars), or high fat diet (HFD, red bars) on gestational day 10 (*n* = 6 in both CD and HFD groups). Mean ± SEM. * = *P* < 0.05 for CD vs. HFD. (B) Serum leptin concentration (ng/ml) on gestational day 0 and 10 of pregnant rats on CD (*n* = 9 and 6, blue bars), or HFD (*n* = 9 and 6, red bars). Mean ± SEM. * = *P* < 0.05 for CD vs. HFD. (C) Serum adiponectin concentration (μg/ml) on gestational day 0 and 10 of pregnant rats on CD (*n* = 9 and 6, blue bars), or HFD (*n* = 9 and 6, red bars). Mean ± SEM. (D) Serum resistin concentration (ng/ml) on gestational day 0 and 10 of pregnant rats on CD (*n* = 9 and 6, blue bars), or HFD (*n* = 9 and 6, red bars). Mean ± SEM. * = *P* < 0.05 for CD vs. HFD.

Maternal serum concentrations of the branched-chain amino acids valine, isoleucine, and leucine were higher on day 10 in HFD rats than in CD rats (Figure 2(A)).

Maternal leptin concentrations in serum of HFD rats were higher than in CD rats on both day 0 and 10 of gestation (Figure 2(B)). There were, however, no differences between HFD and CD rats with regard to maternal adiponectin (Figure 2(C)) and maternal resistin concentrations (Figure 2(D)) on either gestational day.

### Liver components

Total lipids were higher in the HFD livers compared with the CD livers at day 0, whereas the TAG concentrations in the HFD group were increased on day 0 and 20 compared with the corresponding CD pregnant rats. The liver cholesterol concentration was higher in the HFD 0 rats compared with the CD 0 rats. In addition, the concentration of phospholipids was higher in the HFD 20 rats compared with the CD 20 rats (Table 2).

**Table 2.** Liver lipid/liver fatty acid concentrations in day-0, day-10, day-20 pregnant rats on control diet (*n* = 9, 6, 8), or high-fat diet (*n* = 9, 6, 15).

Diet and day of pregnancy	CD 0	Cd 10	Cd 20	HFd 0	HFd 10	HFd 20
Total lipids (mg/g)	47 ± 2	41 ± 4	43 ± 2	98 ± 16^d^	59 ± 5^a^	59 ± 3^b^
TAG (mg/g)	5 ± 1	7 ± 2	8 ± 2	27 ± 8^d^	12 ± 4^a^	16 ± 2^b^^,^^d^
Cholesterol (mg/g)	1.6 ± 0.1	2.4 ± 0.4^a^	1.9 ± 0.1	3.0 ± 0.3^d^	1.8 ± 0.1^a^	2.3 ± 0.2^b^
Phospholipids (mg/g)	10.2 ± 0.2	11.6 ± 1.6	11.1 ± 1.0	9.7 ± 0.4	14.2 ± 0.4^a^	13.7 ± 0.8^b^^,^^d^
Sum SFA	10.9 ± 0.3	13.3 ± 1.0	14.6 ± 0.7	21.2 ± 4.4^d^	15.0 ± 0.8	14.3 ± 1.0^b^
14:0 MA	0.10 ± 0.01	0.16 ± 0.02	0.45 ± 0.03^b^^,^^c^	0.16 ± 0.04	0.10 ± 0.02	0.11 ± 0.01^d^
16:0 PA	4.9 ± 0.2	6.9 ± 0.8	8.3 ± 0.5^b^	10.4 ± 2.4^d^	7.3 ± 0.9	7.0 ± 0.6^b^
18:0 SA	5.7 ± 0.1	6.0 ± 0.3	5.6 ± 0.2	10.5 ± 2.8^d^	7.4 ± 0.3	6.9 ± 0.5^b^
Sum MUFA	3.9 ± 0.3	5.9 ± 1.4	8.0 ± 0.4	10.7 ± 2.8^d^	7.0 ± 1.1	6.8 ± 0.8^b^
18:1 (n-9) OA	3.3 ± 0.3	4.9 ± 1.1	6.5 ± 0.3	10.2 ± 2.7^d^	6.7 ± 1.1	6.4 ± 0.8^b^
Sum n-6 FA	10.6 ± 0.5	10.5 ± 0.6	8.9 ± 0.6	22.1 ± 4.3^d^	15.9 ± 1.1	14.3 ± 1.6^b^^,^^d^
18:2 (n-6) LA	4.1 ± 0.4	4.1 ± 0.3	3.8 ± 0.4	11.9 ± 3.1^d^	7.2 ± 1.2^a^	6.3 ± 0.9^b^
18:3 (n-6) GLA	0.09 ± 0.00	0.13 ± 0.05	0.08 ± 0.00	0.22 ± 0.04^d^	0.13 ± 0.02	0.12 ± 0.01^b^
20:4 (n-6) AA	5.8 ± 0.2	5.3 ± 0.2	4.8 ± 0.2	7.8 ± 0.6^d^	7.0 ± 0.2^d^	6.3 ± 0.6^b^^,^^d^
Sum n-3 FA	2.1 ± 0.1	2.3 ± 0.2	2.4 ± 0.1	3.2 ± 0.4^d^	2.7 ± 0.1	2.6 ± 0.1^b^
18:3 (n-3) ALA	0.09 ± 0.02	0.11 ± 0.02	0.13 ± 0.03	0.30 ± 0.11^d^	0.18 ± 0.03	0.15 ± 0.02^b^
22:5 (n-3) DPA	0.38 ± 0.02	0.31 ± 0.07	0.22 ± 0.02	0.65 ± 0.10^d^	0.36 ± 0.03^a^	0.29 ± 0.04^b^
22:6 (n-3) DHA	1.4 ± 0.1	1.6 ± 0.1	1.6 ± 0.1	2.1 ± 0.2^d^	2.1 ± 0.1^d^	1.9 ± 0.1

Fatty acid concentrations are expressed as mg/g. Mean ± SEM.

a *P* < 0.05, day 0 versus day 10 for each group.

b *P* < 0.05, day 0 versus day 20 for each group.

c *P* < 0.05, day 10 versus day 20.

d *P* < 0.05, CD versus HFD at the corresponding day of pregnancy.

CD: control diet; HFD: high-fat diet.

The sum of SFA was increased in HFD 0 compared with CD 0 livers, and the same pattern was found with regard to 16:0 PA and 18:0 SA. The sum of MUFA and OA (18:0 n-9) were higher in the HFD 0 compared with CD 0 (Table 2).

The sum of PUFA n-6 FAs was higher in HFD 0 and 20 rats compared with the CD 0 and 20 rats. The HFD 0 liver concentrations of LA (18:2 n-6) and GLA (18:3 n-6) were higher than those in the CD 0 rats. In addition, the concentration of AA (20:4 n-6) was increased in the HFD livers at all time points measured. The sum of PUFA n-3 FAs was increased in HFD 0 rat livers compared with CD 0 rat livers. Hepatic concentrations of ALA (18:3 n-3) and DPA (22:5 n-3) were increased in HFD 0 livers compared with CD 0. Furthermore, the concentration of DHA (22:6 n-3) was increased both in HFD 0 and 10 livers compared with the corresponding CD livers (Table 2).

### Adipose tissue

Sums of SFA and of MUFA were higher in the HFD group compared with the corresponding CD group on day 0, whereas the opposite was found on gestational day 20 (Table 3).

**Table 3.** Adipose tissue fatty acid concentration (mg/g) in day-0 or day-20 pregnant rats on control diet (*n* = 9, 8), or high-fat diet (*n* = 9, 15).

Diet and day of pregnancy	CD 0	Cd 20	HFd 0	HFd 20
Sum SFA	273 ± 5	374 ± 17^a^	302 ± 23^b^	257 ± 11^a^^,^^b^
14:0 MA	12.7 ± 0.3	22.2 ± 2.0^a^	14.8 ± 1.4	11.0 ± 0.6^a^^,^^b^
16:0 PA	230 ± 5	313 ± 13^a^	229 ± 20	196 ± 9^b^
18:0 SA	27 ± 1	39 ± 3^a^	56 ± 4^b^	48 ± 2^a^^,^^b^
Sum MUFA	332 ± 7	476 ± 23^a^	434 ± 27^b^	366 ± 15^a^^,^^b^
18:1 (n-9) OA	268 ± 6	376 ± 17^a^	386 ± 24^b^	329 ± 13^a^^,^^b^
Sum n-6 FA	211 ± 6	180 ± 8 ^a^	224 ± 12	197 ± 7^a^
18:2 (n-6) LA	202 ± 6	170 ± 8^a^	213 ± 11	184 ± 7^a^
18:3 (n-6) GLA	1.2 ± 0.03	1.2 ± 0.1	0.7 ± 0.1^b^	0.9 ± 0.1^b^
20:4 (n-6) AA	4.2 ± 0.2	5.1 ± 0.6	4.4 ± 0.4	6.0 ± 0.3^a^
Sum n-3 FA	14 ± 1	21 ± 1^a^	11 ± 1^b^	13 ± 0.4^a^^,^^b^
18:3 (n-3) ALA	11.6 ± 0.5	17.0 ± 1.1^a^	8.6 ± 0.7^b^	9.1 ± 0.3^b^
22:5 (n-3) DPA	0.6 ± 0.04	0.00^a^	0.7 ± 0.1	0.00^a^
22:6 (n-3) DHA	1.0 ± 0.1	1.4 ± 0.2^a^	0.7 ± 0.1	1.2 ± 0.1^a^

Values are mean ± SEM.

a *P* < 0.05, day 0 versus day 20 for each group.

b *P* < 0.05, CD versus HFD at the corresponding day of pregnancy.

CD: control diet; HFD: high-fat diet.

The sum of PUFA n-6 FAs decreased between day 0 and 20 of pregnancy in both groups. Likewise, the concentrations of LA (18:2 n-6) decreased from day 0 to day 20 in both groups. Adipose tissue concentrations of GLA (18:3 n-6) were lower in both the HFD 0 and HFD 20 groups compared with the corresponding CD groups. In addition, the concentrations of AA (20:4 n-6) increased from day 0 to day 20 in the HFD group.

The sum of PUFA n-3 FAs increased from day 0 to day 20 of pregnancy in both groups. In addition, the sum of PUFA n-3 FAs was decreased in HFD rats compared with CD rats at both gestational days. The concentrations of ALA (18:3 n-3) were decreased compared with CD concentrations at both day 0 and 20 of pregnancy. In the CD group, the ALA concentrations increased from day 0 to day 20. The DPA (22:5 n-3) concentration diminished from day 0 to day 20, to the extent that the levels were non-detectable on day 20, in both groups. The adipose tissue concentrations of DHA (22:6 n-3) increased from day 0 to day 20 in both groups (Table 3).

### Gene expression

**Figure 3.**
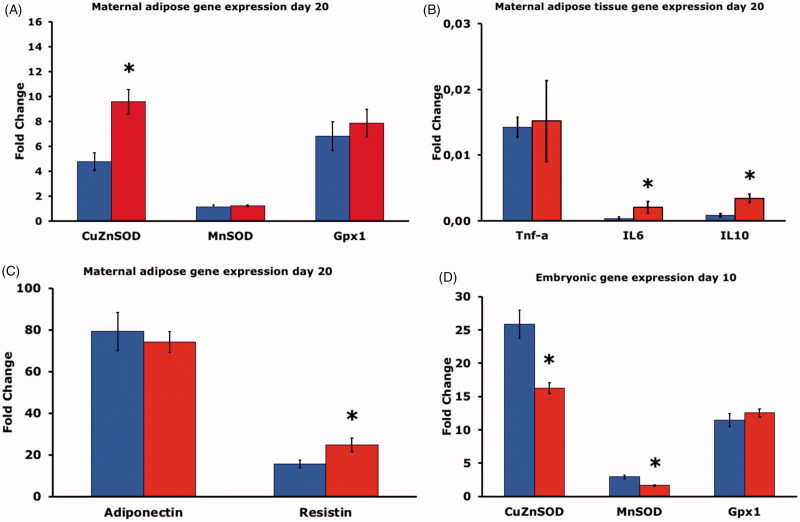
(A) Gene expression of CuZnSOD, MnSOD and Gpx1 in adipose tissue of pregnant rats on control diet (CD, *n* = 8, blue bars), or high fat diet (HFD, *n* = 15, red bars) on gestational day 20. Mean ± SEM. * = *P* < 0.05 for CD vs. HFD. (B) Gene expression of TNF-α, IL-6 and IL-10 in adipose tissue of pregnant rats on CD (*n* = 8 blue bars), or HFD (*n* = 15, red bars) on gestational day 20. Mean ± SEM. * = *P* < 0.05 for CD vs. HFD. (C) Gene expression of adiponectin and resistin in adipose tissue of pregnant rats on CD (*n* = 8, blue bars), or HFD (*n* = 15, red bars) on gestational day 20. Mean ± SEM. * = *P* < 0.05 for CD vs. HFD. (D) Gene expression of CuZnSOD, MnSOD and Gpx1 on gestational day 10 in embryos of six pregnant rats on CD (blue bars, *n* = 30 embryos), or in embryos of six pregnant rats on HFD (red bars, *n* = 30 embryos). Mean ± SEM. * = *P* < 0.05 for CD vs. HFD.

The expression of CuZnSOD in adipose tissue was higher in the HFD group, whereas neither the expression of MnSOD nor Gpx1 differed between the two groups (Figure 3(A)). Concerning cytokines, the expression of TNF-α did not differ between the two groups, but the expression of both IL-6 and IL-10 was higher in adipose tissue of the HFD group than in the CD group (Figure 3(B)). The expression of adiponectin did not differ between the two groups, whereas the expression of resistin was higher in the HFD than in CD rats (Figure 3(C)).

When the mRNA levels in day-10 embryos were measured, we found decreased CuZnSOD and MnSOD gene expression in embryos from HFD rats compared to CD rat embryos. There were no differences in embryonic Gpx1 expression between the groups (Figure 3(D)).

### Fetal outcome

**Figure 4.**
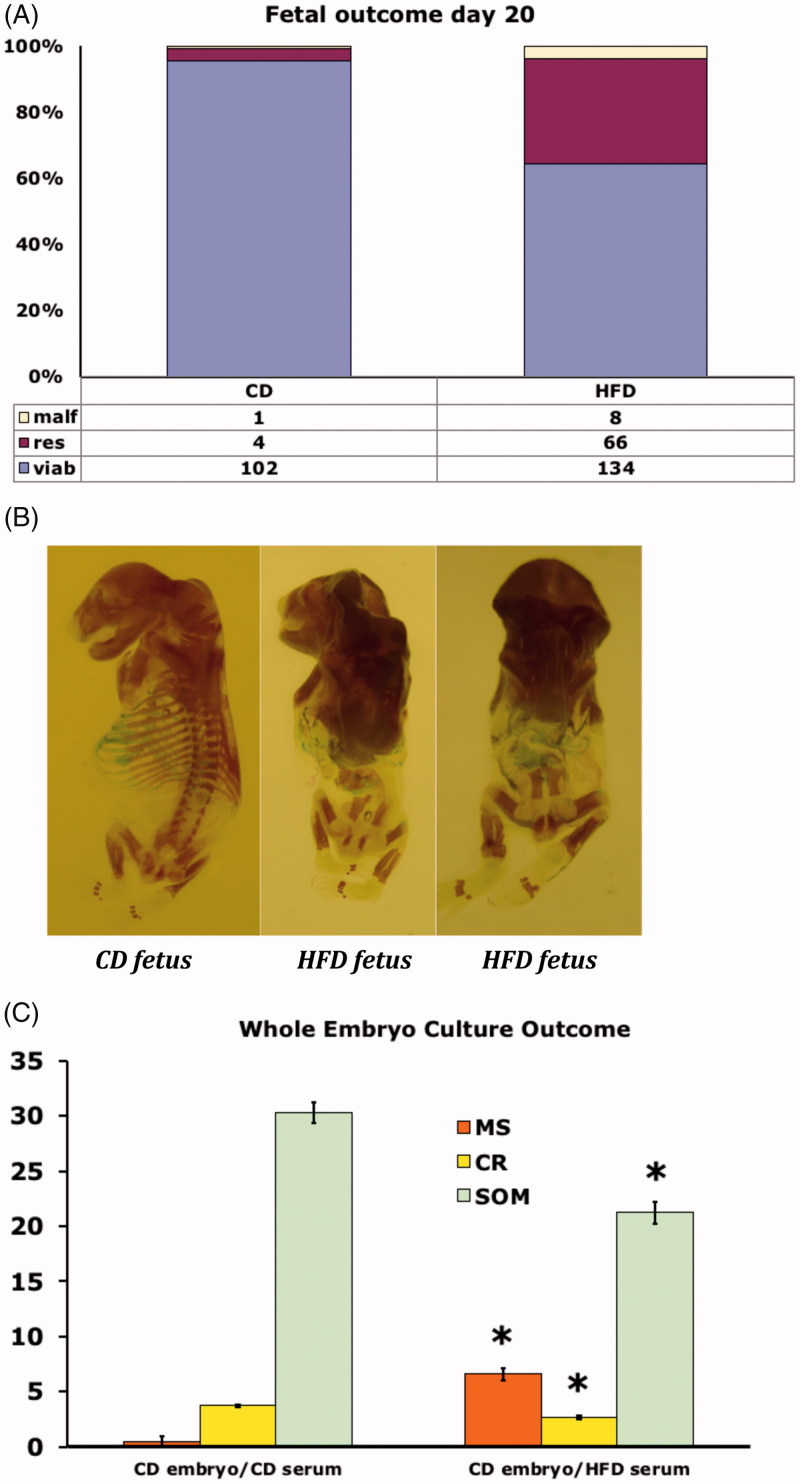
(A) Fetal outcome of rats on control diet (CD, *n* = 8, left bar), or high fat diet (HFD, *n* = 15, right bar) on gestational day 20. Number and proportion of malformed (malf), resorbed (res) and viable (viab) fetuses. (B) Alizarin Red and Alcian Blue stained malformed fetuses from rats on control diet (CD) (left fetus), or high fat diet (HFD) (middle and right fetus). All fetuses show lack of tail, and the two HFD-fetuses (middle and right panel) also demonstrate decreased general ossification. (C) Outcome in CD embryos cultured for 48 h in serum from pregnant rats on either control diet (CD), or in serum from rats on high fat diet (HFD). 20 CD embryos were cultured in CD serum, and 20 CD embryos were cultured in HFD serum. Malformation score (MS), crown-rump length (CR) and somite number (SOM). Mean ± SEM. * = *P* < 0.05 for CD vs. HFD.

The offspring of HFD and CD rats at pregnancy day 20 were studied with regard to fetal and placental weight, malformations, and resorptions. There were no differences in fetal and placental weight (data not shown), whereas we found malformations in 3.8% of the HFD offspring compared with 0.9% in the CD offspring (Figure 4(A)). The malformations were tail and ossification defects (resembling human caudal regression syndrome, cf. Figure 4(B)). Furthermore, we also found resorptions in 32% of the HFD offspring compared with 3.7% in the CD offspring (Figure 4(A)).

### Whole-embryo culture

Day-9 CD embryos were cultured *in vitro* and harvested after 48 h, corresponding to gestational day 11 *in vivo*. We found an increased malformation score (reflecting open neural tube, somatic malrotation, and heart defects) in embryos cultured in serum of day-20 pregnant HFD rats compared with embryos cultured in serum of day-20 pregnant CD rats (Figure 4(C)). Furthermore, we found growth disturbances (decreased crown–rump length, and somite number) in embryos cultured in HFD serum compared with embryos cultured in CD serum (Figure 4(C)).

## Discussion

The most important findings in the present study were that a high-fat diet during pregnancy in rats alters several maternal plasma/serum components and causes marked fetal maldevelopment. This was further corroborated by the demonstration of an enhanced teratogenic potential of HFD maternal serum used in WEC.

HFD rats ingested less food than CD rats, which resulted in both groups consuming the same number of calories (11,12). This agrees with previous reports in rats (28) and suggests that the disturbed embryonic and fetal development in the offspring of HFD rats is the result of the type of dietary FA composition rather than a higher maternal energy intake (29–31). The alterations in maternal HFD plasma/serum were found in all types of nutrients—carbohydrates, proteins, and lipids. Thus, we found increased concentrations of glucose, branched-chain amino acids, and β-hydroxybutyrate in HFD maternal plasma/serum.

In the HFD rats we also found decreased plasma concentrations of TAG and cholesterol, concomitant with decreases of most plasma PUFAs, which fits with the fact that most PUFAs in maternal plasma are carried associated to different lipoproteins in their esterified form both in humans (32) and in rats (33). The HFD rats had decreased plasma concentrations of the n-6 PUFA LA and increased concentration of AA. Also, the n-3 PUFA ALA was decreased.

The increased expressions of IL-6 and IL-10 in the adipose tissue may suggest an ongoing inflammation in the HFD rats. A postulated enhanced secretion of IL-6 by HFD adipose tissue would contribute to chronic inflammation and cause increased C-reactive protein (CRP) production, conditions that may affect the development of the embryo/fetus. In separate studies, we also evaluated CRP effects in adipose tissue by immuno-staining for CD68 and found enhanced macrophage infiltration in HFD rats (Wentzel et al., unpublished), corroborating the impression of an inflammatory state in the HFD rats.

The decreased plasma concentrations of several PUFA n-3 FAs on gestational days 0 and 20 suggest a decreased transfer of PUFA n-3 FAs to the embryo and fetuses during HFD pregnancy (34). Several of the long-chain polyunsaturated FAs (LCPUFAs) have been shown to affect bone cells, i.e. osteoclasts and osteoblasts, via various cellular signaling pathways or growth factors, thereby affecting bone formation, resorptions, and bone density in animals and humans (35). Furthermore, with regard to the reported alterations in both chondrogenesis and osteogenesis caused by diminished PUFA n-3 FAs (36–38), the possibility of a PUFA n-3 FA deficiency-mediated teratogenic insult may be considered, since it is known that maternal LCPUFAs play an important role in fetal development (39).

In contrast to the findings of lowered lipids in HFD maternal plasma, the hepatic concentrations of total lipids, TAG, and cholesterol, as well as most individual FAs were higher in the pregnant HFD rats than in the pregnant CD rats, findings that are in concert with previous reports in HFD-fed rodents (29). Evidently, the hypotriglyceridemia in HFD plasma can be related to increased lipid content in the HFD liver. This accumulation of lipids appears to be a specific hepatic effect rather than adipose tissue process, since the FA profiles found in maternal HFD adipose tissue mainly coincides with the profiles of maternal HFD plasma.

It has been shown that HFD decreases hepatic *de novo* lipogenesis (40) and also decreases the very-low-density lipoprotein secretion rate (40,41). The latter may be the main factor behind the increased liver TAG and cholesterol content, as well as the decreased plasma TAG found in the HFD rats of the present study. Another possibility contributing to the decreased plasma TAG concentrations found in our HFD rats could be an increase in adipose tissue lipoprotein lipase (LPL) activity, which is thought to be an important factor for the removal of TAG from plasma (42). However, we could not detect any change in adipose tissue LPL activity in our HFD rats compared to the activity in CD rats (data not shown). Although plasma post heparin LPL has been shown by Xue et al. to be increased in rats fed HFD for 2 weeks, their plasma TAG removal was actually decreased, and they were still hypertriglyceridemic without change in hepatic TAG secretion (43). It is therefore proposed that the low plasma concentrations of TAG and of most PUFAs in our HFD rats are the result of their retention in the maternal liver. It cannot be excluded that other factors may contribute to such adverse effects of HFD on embryonic-fetal development, i.e. insulin resistance, high levels of branched-chain amino acids and β-hydroxybutyrate, as well as oxidative stress. The higher plasma glucose concentration in the presence of unchanged insulin levels, and increased expression of resistin, IL-6, and IL-10 in adipose tissue of the pregnant HFD rats, suggests an imminent insulin-resistant condition (Supplementary Figure 2, available online). It has been shown previously that HFD causes insulin resistance in pregnant rats, which may alter fetal growth (16). The increased serum concentrations of branched-chain amino acids in our pregnant HFD rats may be the result of a decreased catabolism from a high fat intake, as previously proposed in human subjects (44). The increased branched-chain amino acid concentrations could also influence the embryo-fetal development in the HFD pregnancies as previously suggested in experimental studies of diabetes pregnancy (45,46). In line with this reasoning, a strong synergistic association has been suggested between lipids and branched-chain amino acids promoting metabolic disease (16).

Furthermore, the increased leptin concentration in HFD rats at pregnancy days 0 and 10, together with other metabolic alterations, may be the cause of embryo-fetal maldevelopment and skeletal malformations in the HFD rats (38,47). HFD indeed hampered the embryo-fetal development, as evidenced by increased resorption and malformation rates on gestational day 20. In previous studies, we have estimated the malformation rate in early HFD pregnancy to be around 10% (gestational day 11, *in vivo*, data not shown). In the present study, we found around 4% malformations (mainly skeletal anomalies) on gestational day 20, which may indicate that a number of malformed HFD embryos die *in utero* in late pregnancy and present themselves as resorptions on gestational day 20.

The gene expression of CuZnSOD was increased in maternal HFD adipose tissue, supporting the presence of oxidative stress in tissues involved in adipogenesis (48). In addition, the decreased HFD embryonic gene expression of antioxidant enzymes, both CuZnSOD and MnSOD found here, also suggests that HFD caused embryonic oxidative stress, possibly mediated by embryonic hypoxia (49). This fits with the findings of oxidative stress induced by HFD in other studies (50), which, indeed, may challenge the intrauterine development (17).

In this context, it is worthy of note that serum from day-20 pregnant HFD rats, in itself, is able to hamper embryonic development during only 48 h of exposure, as shown in WEC experiments. Two major differences between HFD and CD plasma/serum are increased concentrations of β-hydroxybutyrate and branched-chain amino acids, both of which have been shown to be teratogenic in WEC experiments (45,46). The possible teratogenic effect of the increased leptin concentration, and decreased concentrations of SFA, MUFA, and n-6 PUFA, of the HFD plasma will be subject to future analysis.

In conclusion, the present study shows that HFD in our rat model alters the maternal metabolic state, increases fetal resorptions *in vivo*, and increases the rate of fetal-embryonic malformations both *in vivo* and *in vitro*. Moreover, HFD decreases embryonic CuZnSOD and MnSOD gene expression and increases gene expression of CuZnSOD, IL-6, IL-10, and resistin in HFD maternal adipose tissue. We also found that HFD causes retention of essential FAs in the maternal liver, which may be responsible for their decreased availability to the embryo-fetus, actively contributing to its short- and long-term adverse consequences. These findings suggest that the metabolic disturbances in HFD-induced maternal obesity have profound adverse developmental effects in the offspring.

## Supplementary Material

Supplemental Material
