# Supplementary material for: High-fat diet in pregnant rats and adverse fetal outcome
Source: Ups J Med Sci. 2019 May 7;124(2):125–34. doi: 10.1080/03009734.2019.1604588 (PMC6567025; doi:10.1080/03009734.2019.1604588)
Supplement: Supplemental Material [file IUPS_A_1604588_SM0896.zip › IUPS_Sup_mat/Suppl Table 1.docx]

*Supplementary Table 1.*

| **Fatty acid** | **CD** | **HFD** | **HFD/CD ratio** |
| --- | --- | --- | --- |
| 14:0 MA | 0.04 | 3.79 | **94.8** |
| 16:0 PA | 5.07 | 66.4 | **13.1** |
| 18:0 SA | 0.60 | 35.6 | **59.3** |
| **SUM SFA** | **6.00** | **106.6** |  |
| 16:1 n-­‐7 POA | 0.12 | 6.32 | **52.7** |
| 18:1 n-­‐9 OA | 4.95 | 119.3 | **24.1** |
| **SUM MUFA** | **5.30** | **128.0** |  |
| 18:2 n-­‐6 LA | 16.4 | 87.0 | **5.3** |
| **SUM n-­‐6** | **16.4** | **91.9** |  |
| 18:3 n-­‐3 ALA | 1.60 | 5.98 | **3.7** |
| **SUM n-­‐3** | **1.63** | **7.23** |  |

Fatty acid composition (mg/g) of the control diet (CD, n = 3), or high fat diet (HFD, n = 3), and the ratio between them (HFD/CD). Values are means of the analysis of three samples of each diet
