# Supplementary material for: High-fat diet in pregnant rats and adverse fetal outcome
Source: Ups J Med Sci. 2019 May 7;124(2):125–34. doi: 10.1080/03009734.2019.1604588 (PMC6567025; doi:10.1080/03009734.2019.1604588)
Supplement: Supplemental Material [file IUPS_A_1604588_SM0896.zip › IUPS_Sup_mat/Suppl Table 2.docx]

*Supplementary Table 2.* Oligonucleotide primer sequences.

G6PDH (F): gTCATgCAgAACCACCTCCT

G6PDH (R): ACATACTggCCAAggACCAC

SOD-1 (F): AAgCggTgAACCAgTTgTg

SOD-1 (R): CCAggTCTCCAACATgCC

SOD-2 (F): ggTggAgAACCCAAAggAgA

SOD-2 (R): AgCAgTggAATAAggCCTgT

Gpx1 (F): TgAgAAgTgCgAggTgAATg

Gpx1 (R): AACACCgTCTggACCTACCA

TNF-a(F): CAgCAgATgggCTgTACCTT

TNF-a(R): CTggAAgACTCCTCCCAggT

IL-6 (F): TgATggATgCTTCCAAACTg

IL-6 (R): gAgCATTggAAgTTggggTA

IL-10 (F): CATgCTCCgAgAgCTgAgggC

IL-10 (R): AATCgATgACAgCgTCgCAgC

Adipo (F): TACCgggCCgTgATggCAgA

Adipo (R): ggTTCCgggAAAgCCCCgTg

Resis (F): gCTgTACCCTgCgggTTggT

Resis (R): TgCgCTCTCCCCATCCCCAT
