# Supplementary figures and images for: High-fat diet in pregnant rats and adverse fetal outcome
Source: Ups J Med Sci. 2019 May 7;124(2):125–34. doi: 10.1080/03009734.2019.1604588 (PMC6567025; doi:10.1080/03009734.2019.1604588)

*Supplementary Figure 1*

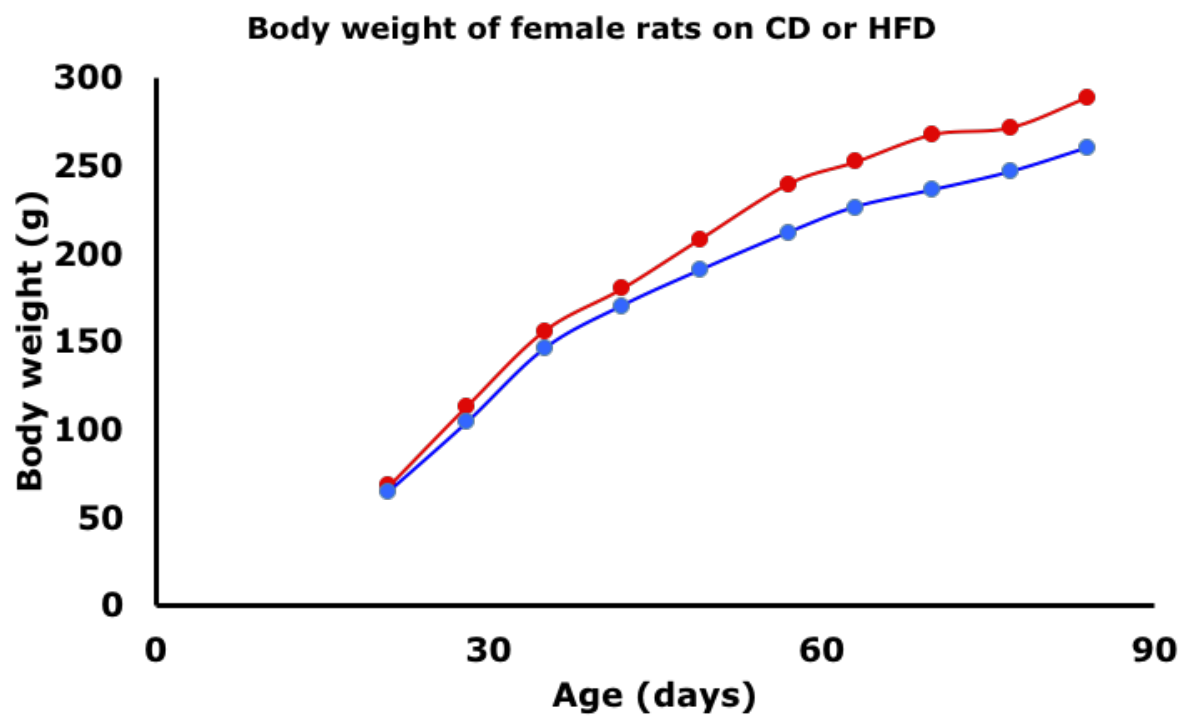

Supplement: Supplemental Material [file IUPS_A_1604588_SM0896.zip › IUPS_Sup_mat/Suppl Fig 1.pdf]

*Supplementary Figure 2*

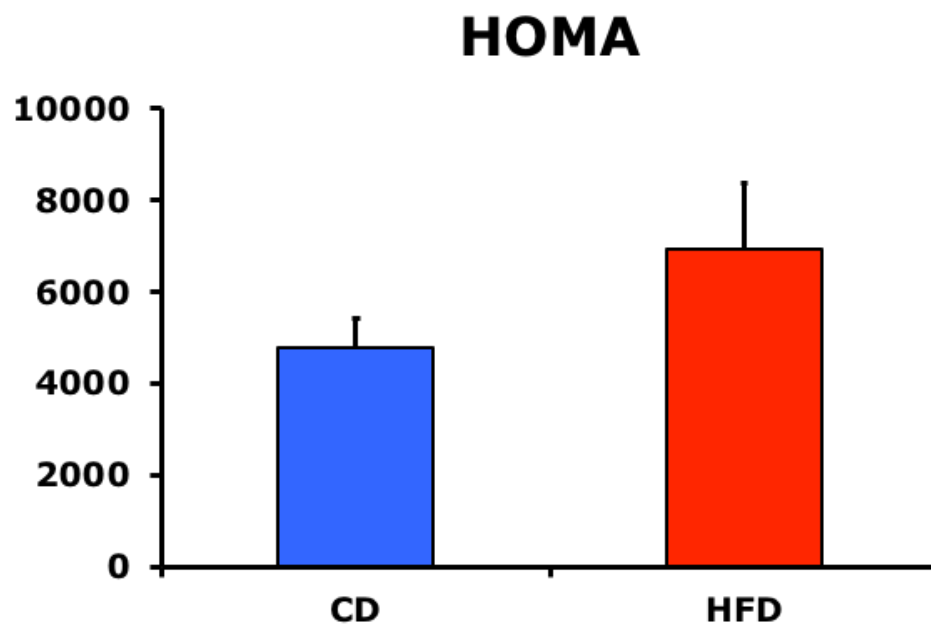

Supplement: Supplemental Material [file IUPS_A_1604588_SM0896.zip › IUPS_Sup_mat/Suppl Fig 2.pdf]
